# Beclin 1 and nuclear factor-κBp65 are upregulated in hepatocellular carcinoma

**DOI:** 10.3892/ol.2013.1307

**Published:** 2013-04-16

**Authors:** KAI-FU KANG, XIAO-WEI WANG, XIAO-WU CHEN, ZI-JING KANG, XIN ZHANG, RHONDA R. WILBUR, FENG CHENG, SHU-FENG ZHOU

**Affiliations:** 1 Departments of Pathology, The First People’s Hospital of Shunde, Southern Medical University, Shunde, Foshan, Guangdong 528300, P.R. China;; 2 General Surgery, The First People’s Hospital of Shunde, Southern Medical University, Shunde, Foshan, Guangdong 528300, P.R. China;; 3 Department of Pharmaceutical Sciences, College of Pharmacy, University of South Florida, Tampa, FL 33612, USA

**Keywords:** beclin 1, nuclear factor-κB, immunohistochemistry, *in situ* hybridization, hepatocellular carcinoma

## Abstract

There are no sensitive and specific biomarkers that aid in the clinical diagnosis and prognosis of hepatocellular carcinoma (HCC). The aim of the present study was to determine the mRNA and protein expression levels of beclin 1 (BECN1) and nuclear factor-κB (NF-κB)p65 in patients with HCC, to evaluate their value as potential diagnostic and prognostic biomarkers. Immunohistochemistry and *in situ* hybridization were used to detect the expression of hepatic BECN1 and NF-kBp65 in patients with HCC, hepatitis B virus (HBV) or cirrhosis, as compared with the expression levels in healthy subjects. The expression level of the BECN1 protein in the HCC tissue was significantly high compared with that in the cirrhotic, hepatitis and normal tissues. The expression of the BECN1 protein in the hepatitis tissue was significantly high compared with that of the cirrhotic and normal tissues. The expression of the *BECN1* mRNA in the cancer tissue was significantly high compared with that of the cirrhotic and normal tissues, and the expression of the *BECN1* mRNA in the hepatitis tissue was significantly higher than that of the cirrhotic and normal tissues. The expression of the NF-κBp65 protein in the cancer tissue was significantly high compared with that of the cirrhotic, hepatitis and normal tissues. The expression of the *NF-κBp65* mRNA in-the cancer tissue was significantly high compared with that of the cirrhotic, hepatitis and normal tissues. BECN1 expression was positively correlated with NF-κBp65 expression in HCC. The abnormal expression of BECN1 and NF-κBp65 was closely associated with the development of HCC. Finally, a search in GeneGo pathway database observed a link between BECN1 and NF-κBp65 through multiple proteins. These results indicate that BECN1 and NF-κBp65 are upregulated in HCC, and that they may serve as useful biomarkers for HCC.

## Introduction

Hepatocellular carcinoma (HCC) is the third leading cause of cancer-related mortality worldwide and is commonly secondary to chronic hepatitis (1,2). Despite recent therapeutic advances, HCC malignancy continues to be a significant cause of cancer-related morbidity and mortality, and it is generally associated with a poor prognosis. The HCC five-year survival rate is 25–39% post-surgery, with systemic therapy using cytotoxic agents providing only marginal benefits (3). Multiple pathogenic factors, including infection with the hepatitis B and C viruses (HBV and HCV) and the subsequent multistage pathogenesis of HCC have been studied extensively. In addition, recent advances in molecular genetics have identified a large number of activated or suppressed genes that may have significant involvement in the process of hepatocarcinogenesis (4); however, the mechanisms by which these factors may promote progression to HCC are unclear.

Nuclear factor-κB (NF-κB) is a heterodimeric complex composed of two subunits of the Rel/NF-κB family, as well as other factors, including NF-κB1 (p50), NF-κB2 (p52), c-Rel, RelA/p65 and RelB (5,6). NF-κB exists in the cytoplasm in its inactive form, in association with the IκB regulatory protein. In response to a variety of stimuli, including inflammatory cytokines, oncogenes and viruses (7), the proteome-dependent degradation of IκB promotes the translocation of NF-κB to the nucleus, the binding of NF-κB to the decameric DNA sequences and the transcriptional activation of the target genes (8–10).

Beclin 1 (*BECN1*), the mammalian orthologue of yeast *Atg6/Vps30,* has been mapped to a tumor susceptibility locus ∼150 kb centromeric to *BRCA1* on human chromosome 17q21 (11). It is a coiled-coil protein that has been identified to act as a tumor suppressor (12). BECN1 is significant in the process of vesicle nucleation of autophagy in association with Bcl-2 (an anti-apoptotic protein) (13), which has been identified to be monoallelically deleted in 40–70% of sporadic mammary, ovarian and prostate tumors.

Two study groups have generated BECN1-deficient mice to investigate whether BECN1 acts as a tumor suppressor, and whether loss of BECN1 may contribute to an increased cancer incidence (14,15). The results demonstrated that the loss of BECN1 is correlated with a reduction in autophagic vacuole formation, and those animals with reduced levels of BECN1 exhibited an unpredicted increase in epithelial and hematopoietic malignancies, including HCC. This data led to the conclusion that BECN1 is a haploinsufficient tumor suppressor gene (16). Two mechanisms by which BECN1 haploinsufficiency promotes cancer are impaired autophagy and increased cell proliferation (13). However, the mechanism by which BECN1 modulates cell death in cancer cells remains unclear. The present study was designed to investigate the correlation between NF-κBp65 activation and the expression of BECN1 in patients with HCC.

## Materials and methods

### Ethics statement

This study was approved by the Ethics Committee of The First People’s Hospital of Shunde, Southern Medical University (Shunde, Guandong, China). All protocols were conducted in accordance with the Declaration of Helsinki (1964). Written informed consent was obtained from the patients and their families.

### Human liver samples

All cases were obtained from the Department of Pathology, The First People’s Hospital of Shunde, and comprised patients with diagnoses from January, 2003 to December, 2007. HCC tissue samples were obtained from 50 participants (47 males and 3 females; median age, 56.5 years; age range, 28–71 years). According to the Edmondson grading system, the histopathological analysis revealed 24 well- or moderately-differentiated tumors (grades 1 and 2) and 26 poorly differentiated or undifferentiated tumors (grades 3 and 4). Liver cirrhosis tissue samples were obtained from 30 participants (22 males and eight females; median age, 50.6 years; age range, 25–69 years). Liver tissue samples from patients with HBV were obtained from 30 participants (28 males and two females; median age, 34.8 years; age range, 6–51 years). Hepatic tissue samples were also obtained from deceased, previously healthy, donors. The Streptavidin Peroxidase (SP) Immune Test kit, anti-NF-κBp65 (mouse monoclonal antibody) and anti-BECN1 (rabbit polyclonal antibody) were purchased from Santa Cruz Biotechnology, Inc. (Santa Cruz, CA, USA). The *in situ* hybridization test kit included probes produced by Boster Biological Technology, Ltd., Wuhan, China.

### SP immunohistochemical staining

SP-immunohistochemistry (SP-IHC) was performed according to the manufacturer’s instructions. The liver tissue samples were formalin-fixed, paraffin-embedded and serially sectioned (4-*μ*m thickness). Following deparaffinization and rehydration with graded ethanol, immunohistochemistry was performed. Endogenous peroxidase was quenched with 3% H_2_O_2_ in deionized water for 10 min and then washed with phosphate-buffered saline (PBS) for 5 min. Antigen retrieval was performed using ethyl-enediaminetetraacetic acid (EDTA; pH 8.0) for 3 min in an autoclave at 118°C, followed by cooling to room temperature. Incubation of the sections in 10% normal goat serum for 15 min blocked the non-specific binding sites. The sections were subsequently treated with primary antibody overnight at 4°C and secondary antibody at 37°C for 30 min. This was then followed by 3,3′-diaminobenzidine (DAB) visualization. Following a number of washes, the sections were counter-stained with hematoxylin. The negative control slides were treated with PBS.

### In situ hybridization

*In situ* hybridization was performed according to the manufacturer’s instructions. All equipment and buffers used were treated with diethylpyrocarbonate (DEPC; Sigma-Aldrich, St Louis, MO, USA). The liver tissue samples were formalin-fixed, paraffin-embedded and serially sectioned (6-*μ*m thickness). Following deparaffinization and rehydration with graded ethanol, the tissues were digested with 2 *μ*g/ml pepsin for 15 min at 37°C, washed in PBS for 5 min and post-fixed with 4% paraformaldehyde in PBS for 10 min. Subsequent to being washed with PBS, the slides were incubated with pre-hybridization solution at 55°C for ≥2 h in a humid chamber. The probes were added to each tissue section and hybridized at 55°C for 16 h. The slides were washed twice in 2X saline sodium citrate (SSC) for 10 min at 37°C, twice in 0.5X SSC for 10 min and twice in 0.2X SSC for 10 min at 55°C. The blocking liquid was then added to the sections, which were allowed to set at room temperature for 30 min to block out the non-specific antigen. Subsequently, anti-mouse digoxin was added to the sections and incubated at 42°C for 4 h, then washed with PBS three times for 5 min. Streptavidin-biotin complex (SABC; Sigma-Aldrich) was added to the sections and maintained at room temperature for 30 min. Horseradish peroxidase (HRP; Sigma-Aldrich) was then added to the sections and maintained at room temperature for 30 min, followed by 3-amino-9-ethylcarbazole (AEC) visualization. Subsequent to a number of washes, the sections were counterstained with hematoxylin. Placebo probes were added as a control.

### Semi-quantitative method

The total BECN1 and NF-κBp65 staining scores were calculated as the sum of the percentage of positively stained tumor cells and the staining intensity scores. Two pathologists incorporated a double-blind method to quantify the number of stained cells. The percentage of positively stained cells was then scored as 1 (<5%, negative), 2 (5–25%, sporadic), 3 (25–50%, focal) or 4 (>50%, diffuse). The staining intensity was scored as 1 (no staining), 2 (weakly stained), 3 (moderately stained) or 4 (strongly stained). The percentage of positively stained cells and the staining intensity were determined utilizing the double-blind design. The final BECN1 and NF-κBp65 expression scores were calculated by multiplying the values of the percentage of positively stained cells and the staining intensity scores; these values ranged between 1 and 16. The expression level was defined as follows: − (score of <4), + (score of 4–8), ++ (score of 9–12) or +++ (score of 13–16).

### Pathway analysis

An online search for the pathways related to BECN1 and NF-κBp65 was performed based on the GeneGo database (http://www.genego.com/). The terms used during our search were “BECN1” and “NF-κB”.

### Statistical analysis

The statistical analysis was conducted with SPSS software, version 10.1 (SPSS, Inc., Chicago, IL, USA). Categorical variables were analyzed using the χ^2^ contingency test and the exact probability test. The Spearman’s rank correlation test was utilized to reveal the correlation between BECN1 and NF-κBp65 expression. P<0.05 was considered to indicate a statistically significant difference.

## Results

### Expression of BECN1 in hepatic tissues

**Figure 1:**
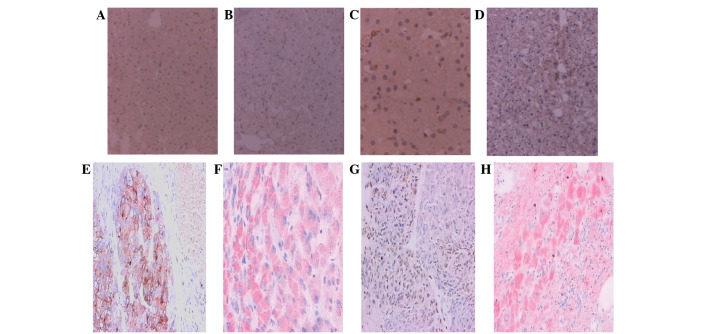
Expression of (A) BECN1 in normal liver; (B) NF-κBp65 in normal liver; (C) BECN1 in liver tissue from patient with HBV; (D) NF-κBp65 in liver tissue from patient with HBV; (E) BECN1 protein in HCC; (F) *BECN1* mRNA in HCC tissue; (G) NF-κBp65 protein in HCC tissue; and (H) *NF-κBp65* mRNA in HCC tissue. (A–E and G) SP immunohistochemistry with DAB staining; (F and H) *in situ* hybridization with AEC staining. Magnification, ×200. BECN1, beclin 1; NF-κBp65, nuclear factor-κB; HBV, hepatitis B virus; HCC, hepatocellular carcinoma.

The BECN1 protein was localized in the cytoplasm of the hepatocytes, stained as brown granules or dots (Fig. 1A, C and E), while *BECN1* mRNA was localized primarily in the cytoplasm of the hepatocytes, appearing as intense red dots distributed in sheets (Fig 1F). The expression rates of BECN1 protein in the HCC, cirrhotic, hepatitis and normal tissues were 78.00, 26.67, 53.33 and 10.00%, respectively (P<0.05; χ^2^=28.34; Table I). The expression of the BECN1 protein in the HCC tissue was significantly higher than that of the cirrhotic, hepatitis and normal tissues (P<0.05; χ^2^=20.39, 5.31 and 14.42, respectively). The expression of the BECN1 protein in the hepatitis tissue was significantly higher than that of the cirrhotic and normal tissues (P<0.05; χ^2^=4.44 and 4.12, respectively).

The positive expression rates of *BECN1* mRNA in the HCC, cirrhotic, hepatitis and normal tissues were 68.00, 23.33, 60.00 and 10.00%, respectively (P<0.05; χ^2^=22.61; Table I). The expression of *BECN1* mRNA in the HCC tissue was significantly higher than that of the cirrhotic and normal tissues (P<0.05; χ^2^=14.97 and 9.27, respectively). The expression of *BECN1* mRNA in the hepatitis tissue was significantly higher than that of the cirrhotic and normal tissues (P<0.05; χ^2^=8.30 and 5.65, respectively).

### Expression of *NF-*κBp65 in hepatic tissues

The NF-κBp65 protein was distributed in the nucleus and/or cytoplasm of the hepatic cells, stained as brown granules or dots (Fig. 1B, D and G), while the *NF*-*κBp65* mRNA was localized mainly in the cytoplasm of the liver cells, resembling intense red dots distributed in sheets (Fig. 1H). The positive expression rates of the NF-κBp65 protein in the HCC, cirrhotic, hepatitis and normal tissues were 74.00, 36.67, 30.00 and 20.00%, respectively (P<0.05; χ^2^=21.42; Table II). The expression of the NF-κBp65 protein in HCC tissue was significantly higher than that of the cirrhotic, hepatitis and normal tissues (P<0.05; χ^2^=10.89, 13.93 and 8.44, respectively).

The positive expression rates of the *NF*-*κBp65* mRNA in the HCC, cirrhotic, hepatitis and normal tissues were 78.00, 33.33, 13.33 and 10.00%, respectively (P<0.05; χ^2^=40.75; Table II). The expression of the *NF-κBp65* mRNA in the HCC tissues was significantly higher than that of the cirrhotic, hepatitis and normal tissues (P<0.05; χ^2^=15.76, 31.54 and 14.42, respectively).

### HCC gene expression and clinical features

The correlations between the immunohistochemistry results and the clinical and pathological findings in the HCC tissues were evaluated. BECN1/NF-κBp65 gene expression was observed to be correlated with HCC tumor size (P<0.05), but not with patient age, Edmondson tumor type, hepatitis B surface antigen (HBsAg) or tumor metastasis (P>0.05; Table III).

### Correlation between BECN 1 and NF-κBp65 expression in HCC

As shown in Table IV, a positive correlation was identified between the *BECN1* and *NF*-*κBp65* mRNA expression levels, with increases in either one promoting increases in the other (Spearman’s correlation rank analysis; P<0.05, r=0.676).

### Pathways for BECN1 and NF-κBp65

A pathway search at the GeneGo website found a comprehensive pathway map for BECN1 and NF-κBp65. It appeared that the myeloid differentiation primary response gene 88 (MYD88) played an important role in the pathway, which linked both proteins through multiple proteins, such as interleukin-1 receptor-associated kinase 1/2 (IRAK1/2), tumor necrosis factor receptor-associated factor 6 (TRAF6), transforming growth factor-β activated kinase 1 (TAK1), c-Jun, toll-like receptor 2 (TLR2) and TLR4.

## Discussion

The present study performed immunohistochemical analyses to determine the expression of the BECN1 and NF-κBp65 proteins in pathogenic and normal hepatic tissues, and to evaluate a potential correlation between BECN1 and NF-κBp65 expression. The expression of the BECN1 and NF-κBp65 proteins in the HCC tissue was significantly higher than that of the cirrhotic, hepatitis and normal tissues. The expression of BECN1 protein in hepatitis tissue was significantly higher than that of cirrhotic and normal tissues, and the BECN1 protein expression was positively correlated with the NF-κBp65 protein expression in the HCC tissue.

An *in situ* hybridization analysis was also performed to detect the expression levels and potential correlations, the results of which were consistent with the immunohistochemical analysis. These results suggested that BECN1 and NF-κBp65 may be important in HCC development. A decreased expression of *BECN1* has been identified in human breast carcinoma, and *BECN1* has been suggested to be a mammalian autophagy gene that may inhibit tumorigenesis (17). This gene has been considered to be a tumor suppressor gene in breast cancer (17,18). The present results demonstrated that the expression of BECN1 mRNA and protein were increased in hepatitis and HCC tissues.

The function of BECN1 in HCC pathogenesis is unclear. The overexpression of BECN1 has been demonstrated to inhibit Sindbis virus replication, reduce central nervous system apoptosis and provide an initial protection against a fatal Sindbis virus infection. BECN1 may therefore be involved in the host defences against a Sindbis virus infection (19). The high expression of BECN1 in hepatitis and HCC tissues may be promoted by viral infection-induced interferon-γ (20). The overexpression of BECN1 may prevent hepatocyte apoptosis, in that HBV infection has been demonstrated to be significant in the development and prognosis of hepatitis, cirrhosis and HCC (21). For cirrhotic tissue, BECN1 (functioning as a tumor suppressor gene) may prevent hepatocyte apoptosis and protect against HBV infection. As autophagy may be involved in either cell death or survival, depending on the cellular context (19,22–24), the increased expression of BECN1 in the hepatitis and HCC tissues may have implications for its unknown biological role. The present study identified that the levels of BECN1 and NF-κBp65 expression in the HCC tissues were not correlated with the clinical and pathological features, including age, Edmondson type, HBsAg or metastasis. However, their expression was enhanced in tumors of a greater size (P<0.05), indicating a potential association with the pathology of HCC.

In numerous cancer cells, the constitutive activation of NF-κB lowers cell sensitivity to apoptotic signaling and consequently to apoptosis, thus favoring neoplastic cell survival (25). In the present study, the expression of NF-κBp65 in cancer tissue was significantly higher than that of the cirrhotic, hepatitis and normal tissues. A significant correlation was demonstrated between BECN1 and NF-κBp65 expression in the HCC tissue, suggesting interactions between the two signaling pathways, which may be mediated via the Bcl-2 family, with apoptosis as the intersection of these two pathways. Under certain conditions, apoptosis and autophagy are able to occur concurrently in the same cell, indicating the involvement of common regulatory mechanisms (26). NF-κB (one of the key regulators of apoptosis) may interact with autophagy. In the present study, BECN1 and NF-κBp65 expression was also observed in the endothelial cells. NF-κB plays a key role in inflammatory disease (10) and may be involved in autophagy, while autophagy itself may also participate in the pathogenesis of inflammation and inflammatory disease.

A comprehensive search from GeneGo pathway database observed a clear link between BECN1 and NF-κBp65 through multiple proteins, including MYD88, IRAK1/2, TRAF6, TAK1, c-Jun, TLR2 and TLR4. Many of these proteins are important regulators of cell proliferation, apoptosis and metabolism and changes in these proteins due to mutations or exposure to risky factors may contribute to the pathogenesis of liver cancer.

In conclusion, the results of the present study indicated that BECN1 and NF-κBp65 are upregulated in primary HCC and may serve as effective biomarkers for the diagnosis of this disease. A search from GeneGo pathway database observed a link between BECN1 and NF-κBp65 through multiple proteins. BECN1 and NF-κBp65 may interact, contributing to the pathogenesis of HCC, however, the precise network that controls the crosstalk between BECN1 and NF-κBp65 is largely unknown. Further studies are required to delineate the functions of BECN1 and its potential correlation with NF-κBp65; this may promote a better understanding of the underlying mechanisms of carcinogenesis and tumor progression in HCC.

## Figures and Tables

**Table I:** Expression of BECN1 in various types of liver tissue.

		BECN1 protein			*BECN1* mRNA		
	
Tissue type	No. of samples	Negative, n	Positive, n	Positive rate (%)	Negative, n	Positive, n	Positive rate (%)
Normal liver	10	9	1	10.00	9	1	10.00
Hepatitis	30	14	16	53.33	12	18	60.00
Cirrhosis	30	22	8	26.67	23	7	23.33
HCC	50	11	39	78.00	16	34	68.00

BECN1, beclin 1; HCC, hepatocellular carcinoma.

**Table II:** Expression of NF-κBp65 in different types of liver tissue.

		NF-κBp65 protein			*NF-κBp65* mRNA		
	
Tissue type	No. of samples	Negative, n	Positive, n	Positive rate (%)	Negative, n	Positive, n	Positive rate (%)
Normal liver	10	8	2	20.00	9	1	10.00
Hepatitis	30	20	9	30.00	26	4	13.33
Cirrhosis	30	19	11	36.67	20	10	33.33
HCC	50	13	37	74.00	11	39	78.00

BECN1, beclin 1; NF-κBp65, nuclear factor-κB; HCC, hepatocellular carcinoma.

**Table III:** Correlation between BECN1/NF-κBp65 protein expression and the clinical features of patients with HCC.

	BECN1 expression				NF-κBp65 expression			
	
Characteristics	Negative, n	Positive, n	χ^2^	P-value	Negative, n	Positive, n	χ^2^	P-value
Age (years)								
≥60 (n=16)	4	12	0.00	P>0.05	3	13	0.21	P>0.05
<60 (n=34)	7	27			10	24		
Tumor size (cm)								
≤5 (n=19)	8	11	5.45	P<0.05	9	10	5.60	P<0.05
>5 (n=31)	3	28			4	27		
Edmondson type								
I/II (n=24)	3	21	2.43	P>0.05	2	12	0.67	P>0.05
III/IV (n=26)	8	18			11	25		
HBsAg								
Positive (n=42)	7	35	2.63	P>0.05	12	30	0.26	P>0.05
Negative (n=8)	4	4			1	7		
Metastasis								
Positive (n=14)	1	13	1.44	P>0.05	2	12	0.67	P>0.05
Negative (n=36)	10	26			11	25		

BECN1, beclin 1; NF-κBp65, nuclear factor-κB; HBsAg, hepatitis B surface antigen; HCC, hepatocellular carcinoma.

**Table IV:** Correlation between BECN1 and NF-κBp65 mRNA expression in HCC.

		NF-κBp65, n			
BECN1, n	No. of samples	−	+	++	+++
−	16	10	3	2	1
+	8	1	4	2	1
++	9	0	1	5	3
+++	17	0	1	8	8
Total	50	11	9	17	13

r=0.676, P<0.05. BECN1, beclin 1; NF-κBp65, nuclear factor-κB; HCC, hepatocellular carcinoma;

−, expression score of <4;

+, expression score of 4–8;

++, expression score of 9–12;

+++, expression score of 13–16.

## References

[1] Block TM, Mehta AS, Fimmel CJ, Jordan R (2003). Molecular viral oncology of hepatocellular carcinoma. Oncogene.

[2] El-Serag HB (2011). Hepatocellular carcinoma. N Engl J Med.

[3] Thomas MB, Zhu AX (2005). Hepatocellular carcinoma: the need for progress. J Clin Oncol.

[4] Frenette C, Gish RG (2011). Hepatocellular carcinoma: molecular and genomic guideline for the clinician. Clin Liver Dis.

[5] Baeuerle PA, Baltimore D (1996). NF-kappa B: ten years after. Cell.

[6] Siebenlist U, Franzoso G, Brown K (1994). Structure, regulation and function of NF-kappa B. Annu Rev Cell Biol.

[7] Pikarsky E, Porat RM, Stein I, Abramovitch R, Amit S, Kasem S (2004). NF-kappaB functions as a tumour promoter in inflammation-associated cancer. Nature.

[8] Chan CF, Yau TO, Jin DY, Wong CM, Fan ST, Ng IO (2004). Evaluation of nuclear factor-kappaB, urokinase-type plasminogen activator, and HBx and their clinicopathological significance in hepatocellular carcinoma. Clin Cancer Res.

[9] Karin M, Lin A (2002). NF-kappaB at the crossroads of life and death. Nat Immunol.

[10] Tak PP, Firestein GS (2001). NF-kappaB: a key role in inflammatory diseases. J Clin Invest.

[11] Aita VM, Liang XH, Murty VV, Pincus DL, Yu W, Cayanis E (1999). Cloning and genomic organization of beclin 1, a candidate tumor suppressor gene on chromosome 17q21. Genomics.

[12] Friedman LS, Ostermeyer EA, Lynch ED, Szabo CI, Anderson LA, Dowd P (1994). The search for BRCA1. Cancer Res.

[13] Liang XH, Jackson S, Seaman M, Brown K, Kempkes B, Hibshoosh H, Levine B (1999). Induction of autophagy and inhibition of tumorigenesis by beclin 1. Nature.

[14] Qu X, Yu J, Bhagat G, Furuya N, Hibshoosh H, Troxel A (2003). Promotion of tumorigenesis by heterozygous disruption of the beclin 1 autophagy gene. J Clin Invest.

[15] Yue Z, Jin S, Yang C, Levine AJ, Heintz N (2003). Beclin 1, an autophagy gene essential for early embryonic development, is a haploinsufficient tumor suppressor. Proc Natl Acad Sci USA.

[16] Edinger AL, Thompson CB (2003). Defective autophagy leads to cancer. Cancer Cell.

[17] Liang XH, Yu J, Brown K, Levine B (2001). Beclin 1 contains a leucine-rich nuclear export signal that is required for its autophagy and tumor suppressor function. Cancer Res.

[18] Liang XH, Kleeman LK, Jiang HH, Gordon G, Goldman JE, Berry G (1998). Protection against fatal Sindbis virus encephalitis by beclin, a novel Bcl-2-interacting protein. J Virol.

[19] Qian YW, Wang YC, Hollingsworth RE, Jones D, Ling N, Lee EY (1993). A retinoblastoma-binding protein related to a negative regulator of Ras in yeast. Nature.

[20] Li P, Du Q, Cao Z, Guo Z, Evankovich J, Yan W (2012). Interferon-γ induces autophagy with growth inhibition and cell death in human hepatocellular carcinoma (HCC) cells through interferon-regulatory factor-1 (IRF-1). Cancer Lett.

[21] Alison MR, Nicholson LJ, Lin WR (2011). Chronic inflammation and hepatocellular carcinoma. Recent Results Cancer Res.

[22] Alva AS, Gultekin SH, Baehrecke EH (2004). Autophagy in human tumors: cell survival or death?. Cell Death Differ.

[23] Baehrecke EH (2005). Autophagy: dual roles in life and death?. Nat Rev Mol Cell Biol.

[24] Kroemer G, Jäättelä M (2005). Lysosomes and autophagy in cell death control. Nat Rev Cancer.

[25] Bours V, Dejardin E, Goujon-Letawe F, Merville MP, Castronovo V (1994). The NF-kappa B transcription factor and cancer: high expression of NF-kappa B- and I kappa B-related proteins in tumor cell lines. Biochem Pharmacol.

[26] Jäättelä M (2004). Multiple cell death pathways as regulators of tumour initiation and progression. Oncogene.

